# Pseudoaneurysm of the ascending aorta in a patient with ascending aorta aneurysm and our surgical procedure

**DOI:** 10.1186/1749-8090-8-S1-P3

**Published:** 2013-09-11

**Authors:** K Ergunes, L Yilik, I Peker, N Karahan, Y Besir, A Gurbuz

**Affiliations:** 1 Izmir Katip Celebi University Ataturk Training and Research Hospital, Department of Cardiovascular Surgery, Izmir, Turkey

## Background

The wall of aneurysm in aortic aneurysm is composed of the normal histological component of aorta. Pseudoaneurysm represents a rupture which does not contain the normal histological component of aorta. We described a case of pseudoaneurysm of the ascending aorta in patient with ascending aorta aneurysm.

## Methods

We presented the case of 58-year-old man. He admitted to our hospital with for chest pain. Chest computed tomography showed that the ascending aorta was 55 mm and a pseudoaneurysm was observed about 2 cm of non-coronary cusp. Arterial access to establish cardiopulmonary bypass performed via the right common femoral artery. Venous cannulation was performed with right atrial double-stage cannula. Myocardial protection was achieved with a combination antegradely and retrogradely of isothermic blood cardioplegia.

## Results

Proximal ascending aorta including pseudoaneurysm was resected from about 1.5 cm of coroner cusps and distal ascendin aorta was resected about 2 cm proximal of truncus brachiocephalicus. A 28 mm tube graft was replaced between proximal and distal ascending aorta. The operation and recovery was uneventful.

## Conclusions

This unusual presentation of pseudoaneurysm in patient with ascending aort aneurysm can help to manage similar cases.

